# Spotlight on recent advances in cardiovascular biology

**DOI:** 10.1038/s12276-019-0354-8

**Published:** 2019-12-19

**Authors:** Hyun Kook, Hee Cheol Cho

**Affiliations:** 1 Vascular Remodeling Research Center, Chonnam National University Medical School, Gwangju, 501-746 Republic of Korea; 2 Department of Pharmacology, Chonnam National University Medical School, Gwangju, 501-746 Republic of Korea; 3 Wallace H. Coulter Department of Biomedical Engineering, Georgia Institute of Technology and Emory University, Atlanta, GA USA; 4 Department of Pediatrics, Emory University, Atlanta, GA USA

**Keywords:** Translational research, Mechanisms of disease

Classically, basic cardiovascular research has been delimited to investigating one of the two architectural territories of the heart; the myocardium and the coronary vasculature. Technological advances continue to foster mechanistic insights on homeostatic physiology and pathophysiology of the heart. In parallel, translational efforts in gene therapy^1^ and stem cell biology^2^ have exploited newly-gained concepts toward creating disease-modifying activities in animal models and clinical trials. Together, these advances have blurred the structural boundaries between the myocardium and the circulation, and brought us to appreciate the key signaling pathways that are common to various domains of the heart as well as the fluidity of molecular signals that play opposing roles depending on the spatial and temporal context.

**Fig. 1:**
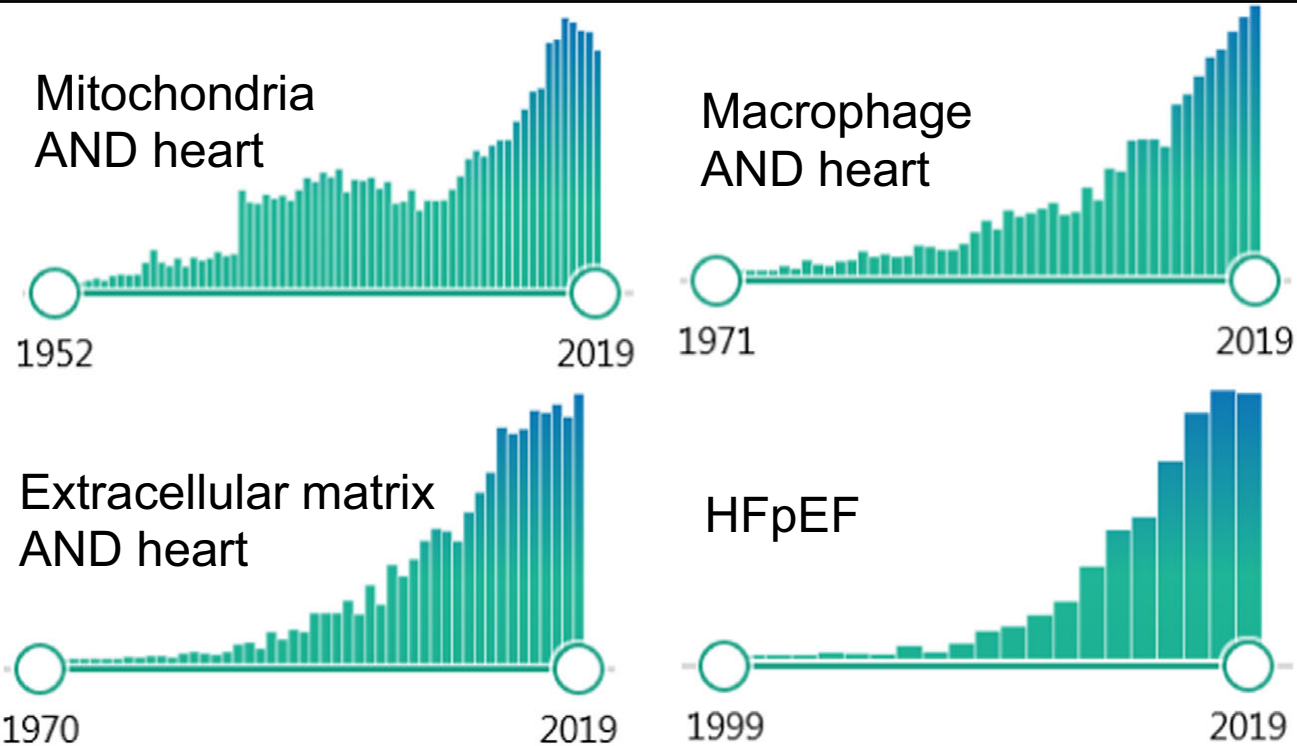
Recent increases in the number of citations on PubMed with regard to four topics. PubMed search on the keywords limited to Title/Abstract returned 4039 results with “(extracellular matrix) AND heart)”, 2,089 results with “(macrophage) AND (heart)”, 10,300 results with “(mitochondria) AND (heart)”, and 2366 results with “heart failure with preserved ejection fraction”.

In this thematic series, four review articles bring spotlights on key conceptual advances in the cardiovascular research. It opens with Kwong and colleagues’ article on mitochondrial metabolism, and strategies to target specifically the mitochondrial reactive oxygen species for better therapeutic outcomes. De Couto connects cardiac regeneration/repair with one of the most dynamic cell types within the heart, the macrophages, and presents approaches to modulating the immune cells’ properties with extracellular vesicles. Kassiri and colleagues highlight the significance of the extracellular milieu in the context of aortic aneurism, and the importance of choosing the correct model to study specific matrix. Finally, Eom and Yoon bring together the molecular concepts and present the-state-of-the art in understanding an elusive form of heart failure that appears to be normal in its ability to pump blood effectively. All four articles underscore topics that are highly relevant now. A quick search on PubMed illustrate nearly an exponential increase in the number of peer-reviewed articles published in recent years on each topic (Fig. 1). What ties the four articles together is the aforementioned idea; understanding principal components of mitochondrial metabolism, macrophage modulation, and extracellular matrix will be key to developing therapeutic approaches to a complex disease such as heart failure.
